# Cardiac involvement in patients with Becker muscular dystrophy: new diagnostic and pathophysiological insights by a CMR approach

**DOI:** 10.1186/1532-429X-10-50

**Published:** 2008-11-04

**Authors:** Ali Yilmaz, Hans-Jürgen Gdynia, Hannibal Baccouche, Heiko Mahrholdt, Gabriel Meinhardt, Cristina Basso, Gaetano Thiene, Anne-Dorte Sperfeld, Albert C Ludolph, Udo Sechtem

**Affiliations:** 1 Division of Cardiology, Robert-Bosch-Krankenhaus, Stuttgart, Germany; 2 Department of Neurology, University of Ulm, Ulm, Germany; 3 Department of Medico-Diagnostic Sciences, University of Padua Medical School, Padua, Italy

## Abstract

**Background:**

Becker-Kiener muscular dystrophy (BMD) represents an X-linked genetic disease associated with myocardial involvement potentially resulting in dilated cardiomyopathy (DCM). Early diagnosis of cardiac involvement may permit earlier institution of heart failure treatment and extend life span in these patients. Both echocardiography and nuclear imaging methods are capable of detecting later stages of cardiac involvement characterised by wall motion abnormalities. Cardiovascular magnetic resonance (CMR) has the potential to detect cardiac involvement by depicting early scar formation that may appear before onset of wall motion abnormalities.

**Methods:**

In a prospective two-center-study, 15 male patients with BMD (median age 37 years; range 11 years to 56 years) underwent comprehensive neurological and cardiac evaluations including physical examination, echocardiography and CMR. A 16-segment model was applied for evaluation of regional wall motion abnormalities (rWMA). The CMR study included late gadolinium enhancement (LGE) imaging with quantification of myocardial damage.

**Results:**

Abnormal echocardiographic results were found in eight of 15 (53.3%) patients with all of them demonstrating reduced left ventricular ejection fraction (LVEF) and rWMA. CMR revealed abnormal findings in 12 of 15 (80.0%) patients (p = 0.04) with 10 (66.6%) having reduced LVEF (p = 0.16) and 9 (64.3%) demonstrating rWMA (p = 0.38). Myocardial damage as assessed by LGE-imaging was detected in 11 of 15 (73.3%) patients with a median myocardial damage extent of 13.0% (range 0 to 38.0%), an age-related increase and a typical subepicardial distribution pattern in the inferolateral wall. Ten patients (66.7%) were in need of medical heart failure therapy based on CMR results. However, only 4 patients (26.7%) were already taking medication based on clinical criteria (p = 0.009).

**Conclusion:**

Cardiac involvement in patients with BMD is underdiagnosed by echocardiographic methods resulting in undertreatment of heart failure. The degree and severity of cardiac involvement in this population is best characterised when state-of-the-art CMR methods are applied. Further studies need to demonstrate whether earlier diagnosis and institution of heart failure therapy will extend the life span of these patients.

## Background

Muscular dystrophy type Becker-Kiener (BMD) and type Duchenne (DMD) represent X-linked genetic diseases related to mutations in the dystrophin gene which is located on chromosome Xp21.1 [1]. BMD has been described to have a prevalence rate of 2.4 per 100,000 and is less common than DMD [2]. While the protein dystrophin is totally absent or dysfunctional in DMD, BMD is characterised by a reduced expression of (possibly dysfunctional) dystrophin. Respiratory failure constitutes the main cause of mortality (mostly until the third decade of life) in DMD, whereas BMD patients demonstrate a later onset and a different as well as slower progression of the disease: BMD patients may survive until the sixth decade of life and cardiomyopathy represents the number one cause of death [3].

Myocardial involvement with the development of dilated cardiomyopathy (DCM) has been described in up to 72% of BMD patients, mainly based on echocardiographic studies [4]. It is believed that dystrophin protein dysfunction, fragility or absence is causing defects in plasma membrane stabilization, in signal and force transduction and in resistance to mechanical stress. Additional mechanical stress may then lead to further disruption of the plasma membrane-sarcomere interaction resulting in cardiomyopathy. DCM is progressive and associated with heart failure, ventricular arrhythmias and is an important cause of morbidity and mortality in these patients [5].

Early diagnosis of cardiac involvement in patients with BMD may be useful, since an early institution of heart failure medication may lead to beneficial ventricular remodelling with improvement in LV systolic function or at least to decelerating progressive dysfunction [6,7]. However, early diagnosis may be difficult using conventional non-invasive methods such as echocardiography as primarily wall motion abnormalities are depicted which characterise the later stages of myocardial involvement. Therefore, cardiovascular magnetic resonance (CMR) may be a promising non-invasive tool for accurate and comprehensive cardiac evaluation of patients with muscular dystrophy (MD) – and in particular BMD – enabling a more timely implementation of appropriate treatment strategies [8-11]. However, no comprehensive and targeted CMR studies have been performed so far on patients with BMD.

With this study, we aimed at 1) comprehensively characterising cardiac disease manifestation in patients with BMD, 2) demonstrating the superiority of CMR compared to conventional echocardiography in cardiac evaluation of patients with BMD and 3) elucidating the in vivo cardiac damage pattern of patients with BMD.

## Methods

### Study design

In a prospective two-center-study, 15 patients with BMD were consecutively ascertained and studied with respect to their neurological disorder at the neuromuscular outpatient clinic of the Department of Neurology, University of Ulm, Germany, followed by a comprehensive cardiological work-up at the Division of Cardiology of the Robert-Bosch-Krankenhaus Stuttgart, Germany. The diagnosis of BMD was confirmed by skeletal muscle biopsy evaluation with immunohistochemical analysis of the dystrophin protein and/or a genetic work-up with screening for dystrophin gene deletion and duplication. Cardiological work-up included physical examination, 12-lead ECG, echocardiography and CMR. Blood samples were obtained for laboratory studies including measurement of total creatine kinase (CK), cardiac troponin-I (TnI), brain-natriuretic-peptide (BNP) and C-reactive-protein (CRP) apart from others. This study was approved by the local ethical committee. Written informed consent was obtained from each patient or his legal guardian before inclusion to this study.

### Echocardiography

Two-dimensional echocardiography was performed on a commercially available system (Vivid 7, GE Healthcare) using a 3.5-MHz transducer with the patient in the left lateral decubitus position. Standard two-dimensional long and short axis views were recorded with the transducer in the apical and parasternal position. Measurements included left ventricular (LV) endsystolic and enddiastolic volumes. Biplane LV ejection fraction (LVEF) was calculated using the Simpson's rule. A semiquantitative global wall motion score index (GWMS) was calculated based on a 16-segment model as recommended by the American Society of Echocardiography [12]: two experienced echocardiographers blinded to the patient's clinical information visually scored wall motion on a 0 to 3 scale for each wall segment (normal = 0, hypokinetic = 1, akinetic = 2, dyskinetic = 3). The GMWS was then calculated as the sum of the myocardial wall motion segment scores divided by the number of segments assessed.

### Cardiovascular magnetic resonance

Electrocardiographic-gated CMR was performed in breath-hold using a 1.5-T system (Magnetom Sonata, Siemens Medical Solutions, Erlangen, Germany). Both cine and late gadolinium enhancement (LGE) short-axis CMR images were prescribed every 10 mm (slice thickness 6 mm) from base to apex. In-plane resolution was typically 1.2 × 1.8 mm. In addition to cine CMR, LGE images were acquired on average five to ten minutes after contrast administration with the use of a segmented inversion recovery gradient echo technique with constant adjustment of inversion time [13]. The contrast dose (Magnevist^®^, Bayer-Schering, Germany) was 0.15 mmol/kg. In addition, fat-saturated and T2-weighted images were obtained to allow differentiation among subepicardial LGE, epicardial fat, and pericardial effusion.

Cine and contrast images were evaluated by two blinded observers as described elsewhere [14]. In brief, endocardial and epicardial borders were outlined on the short-axis cine images. Volumes and EF were derived by summation of epicardial and endocardial contours. The extent of LGE was planimetered applying ImageJ software (National Institutes of Health, Bethesda, Md, USA) on the short-axis contrast images with the use of an image intensity level ≥4 SD above the mean of remote myocardium to define LGE indicative of myocardial damage. In addition, a semiquantitative GWMS was calculated based on a 16-segment model with cine short-axis CMR images similar to echocardiographic analysis.

### Statistical analysis

Data for continuous variables are expressed as median values in addition to minimal and maximal values, whereas data for categorical variables are expressed as the number and the percentage of patients. Comparisons between groups were done using a two-sided unpaired Student's t-test for normally distributed, and Mann-Whitney U test for non-normally distributed continuous variables. For categorical variables we used the chi-square test and Fisher's exact test, where appropriate. Agreement between methods was measured with Kappa statistics. Simple linear regression analyses were performed in order to identify possible determinants of reduced LVEF. The difference between two groups was defined to be statistically significant if the two-sided p-value was < 0.05.

## Results

### Patient characteristics

The patient characteristics are shown in Table 1. The median age of this collective was 37 years with a maximum of 56 years and a minimum of 11 years. Muscle biopsy had been performed in 13 patients with 12 of them demonstrating reduced levels of dystrophin in the immunohistochemical stainings. Genetic analyses for dystrophin gene deletion and duplication mutations were available in 12 patients with eight of them demonstrating DNA mutations. Cardiac involvement indicating early or advanced DCM had been previously diagnosed in seven patients by echocardiography.

**Table 1:** Patient characteristics

**Patient no.**	**Age** **[years]**	**BMI** **[kg/m^**2**^]**	**Age at BMD diagnosis** **[years]**	**Family history for BMD**	**Skeletal muscle biopsy**	**Genetic study**	**Myopathic EMG**	**Established heart disease**	**Current medication**
**1**	49	31	45	-	+	+	+	+	ACE-I + β-BA + Diur.
**2**	56	29	25	-	+	n/a	+	+	β-BA + Diur.
**3**	42	22	15	-	+	-	+	+	ACE-I + Diur.
**4**	51	23	1	-	+	-	+	+	-
**5**	26	22	4	-	+	-	+	-	-
**6**	23	24	1	-	+	+	+	+	-
**7**	37	24	6	-	+	-	+	-	-
**8**	35	23	10	-	+	+	+	+	ACE-I
**9**	26	19	6	-	+	n/a	+	-	-
**10**	43	23	20	-	-	+	+	-	-
**11**	11	23	2	-	+	n/a	+	-	-
**12**	41	25	1	+	+	+	+	-	-
**13**	14	19	0.5	-	+	+	+	-	-
**14**	12	14	6	-	n/a	+	+	-	-
**15**	55	23	33	+	+	+	+	+	-

BMI = body mass index; EMG = electromyogram; ACE-I = angiotensin-converting-enzyme inhibitor; β-BA = beta-blocking agent; Diur. = diuretic; n/a = not applied.

### Laboratory and ECG findings

**Figure 1:**
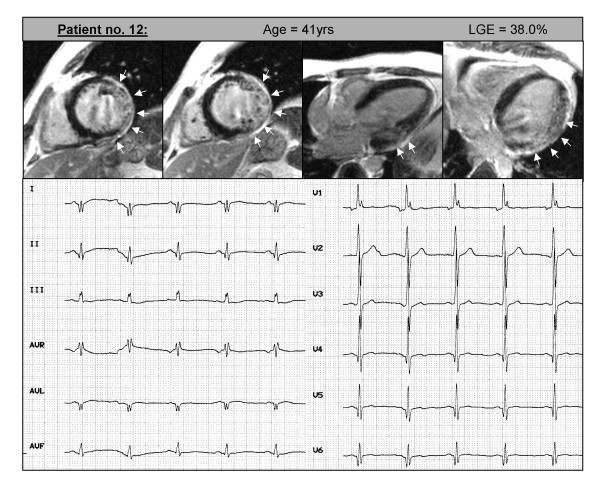
Contrast CMR images of patient no. 12 in the following order (from left to right): two short-axis views, a 3-chamber-view and a 4-chamber view demonstrating a transmural pattern of LGE in the LV free wall representing myocardial damage (white arrows). In addition, this patient presented with ECG changes previously described to be typical in muscular dystrophy (R:S-ratio ≥1.0 in lead V1, a deep Q-wave in leads I, II, aVL, V5-V6 or a complete right bundle branch block).

As expected, all patients had elevated serum CK enzymes, however, all but one patient were TnI-negative suggesting a skeletal muscle origin of these enzymes (Table 2). Interestingly, BNP serum values were elevated only in two patients in spite of depressed LV function (LVEF <60%) in 10 patients as demonstrated by CMR analyses. ECG abnormalities were observed in seven patients (Table 2). However, only four of these seven patients demonstrated those ECG abnormalities previously described to be typical in patients with MD [15] including a R:S-ratio ≥1.0 in lead V1, a deep Q-wave in leads I, II, aVL, V5-V6 or a complete right bundle branch block (Fig. 1).

**Table 2:** Patient findings

**Patient no.**	**Age** **[years]**	**CK** **[U/L]**	**TnI** **[μg/L]**	**CRP >0.5 mg/dL**	**BNP >80 pg/mL**	**Abnormal ECG signs**	**Abnormal Echo findings**	**Abnormal CMR findings**
**1**	49	3609	0.66	No	No	Yes	Yes	Yes
**2**	56	593	0.01	No	Yes	Yes	Yes	Yes
**3**	42	2181	0.01	No	No	No	Yes	Yes
**4**	51	311	0.01	No	No	Yes	Yes	Yes
**5**	26	2570	0.02	No	No	No	No	Yes
**6**	23	310	0.02	No	No	No	No	Yes
**7**	37	564	0.01	No	No	Yes	Yes	Yes
**8**	35	828	0.01	No	No	No	Yes	Yes
**9**	26	3668	0.01	No	No	No	No	Yes
**10**	43	611	0.01	No	No	No	No	Yes
**11**	11	2845	0.01	No	No	No	No	No
**12**	41	315	0.01	No	No	Yes	Yes	Yes
**13**	14	28194	0.01	No	No	No	No	No
**14**	12	6875	0.03	No	No	Yes	No	No
**15**	55	1532	0.01	Yes	Yes	Yes	Yes	Yes

CK = creatinine kinase; TnI = troponin I; CRP = C-reactive protein; BNP = brain-natriuretic protein; ECG = electrocardiogram.

### Echocardiographic results in comparison to CMR results

Abnormal echocardiographic results defined as LVEF<60% and/or visual observation of rWMA were present in eight of 15 (53.3%) patients with all eight patients demonstrating reduced LVEF as well as some rWMA (median GWMS = 0.19; range 0 to 1.19). Interestingly, CMR revealed abnormal findings (defined as LVEF<60% and/or rWMA and/or presence of LGE) in 12 of 15 (80.0%) patients (p = 0.040 compared to echocardiography) with 10 (66.6%) of them having reduced LVEF (median LVEF = 52%; range 27% to 70%; p = 0.16 compared to echocardiography) as well as 9 (64.3%) demonstrating rWMA (median GWMS = 0.19; range 0 to 1.06; p = 0.38 compared to echocardiography). Both techniques revealed rWMA in the same eight patients. However, using echocardiography rWMA was mostly observed in septal wall segments (n = 4) followed by lateral segments (n = 2) while CMR revealed rWMA primarily in the (infero-)lateral segments (n = 6). The kappa-test showed good agreement in the assessment of rWMA with echocardiography compared to CMR when studies without any rWMA were included to the analysis (kappa = 0.86). However, there was only a modest agreement in the assessment of rWMA when studies without rWMA were excluded (kappa = 0.37).

### Myocardial damage imaged with CMR

**Figure 2:**
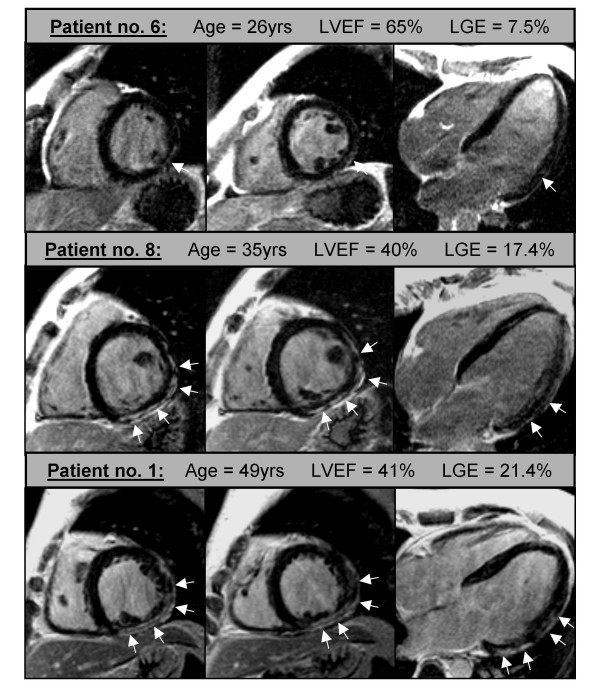
Typical contrast CMR images of three different patients (age 26 years to 49 years) each with two short-axis and one four-chamber view. Subepicardial LGE suggestive of myocardial damage (white arrows) was detected in all three patients in the inferolateral and contiguous lateral wall with age-dependent increase in its extent (7.5% to 21.4%).

Myocardial damage as indicated by LGE imaging was detected in 11 of 15 (73.3%) patients with a median myocardial damage extent of 13.0% (range 0 to 38.0%; Fig. 2). The most common pattern of LGE was a subepicardial to intramural extension in the inferolateral wall (n = 8) followed by an intramural location in septal segments (n = 2) and a transmural pattern in the lateral wall in one patient. A substantial agreement between the presence of myocardial damage as demonstrated by LGE imaging and rWMA was detected by kappa analysis (kappa = 0.69). Interestingly, two patients with preserved systolic LV function also demonstrated subepicardial LGE in the inferolateral wall. Correlation and regression analyses revealed a substantial association between increasing myocardial damage extent measured by LGE imaging and higher age (r^2 ^= 0.770; p < 0.001). The youngest patient demonstrating at least some LGE was found to be 26 years of age and all patients older than 26 years had some myocardial damage. LV systolic function was depressed in the absence of LGE only in one patient aged 23 years.

### Comparison of patients with normal vs. reduced LVEF

**Figure 3:**
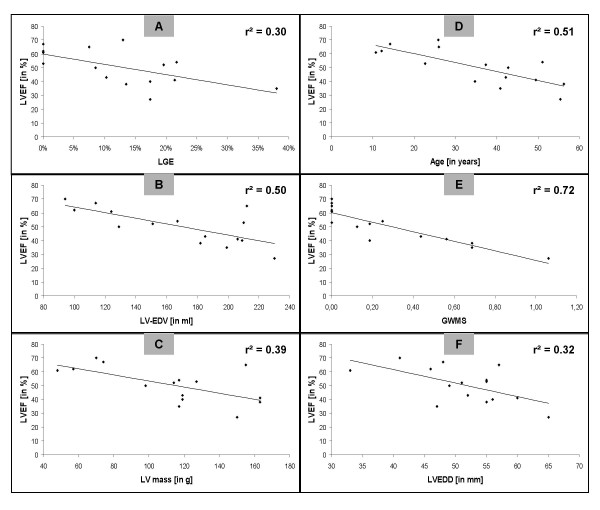
Graphs visualizing correlation analyses comprising all 15 study patients and demonstrating the association between LVEF (measured by CMR analysis) and extent of LGE (panel A), LV-EDV (panel B), LV-mass (panel C), age (panel D), GWMS (panel E) and LVEDD (panel F), respectively.

When patients with normal LVEF (≥60%; n = 5) were compared to those with reduced LVEF (<60%; n = 10), significant differences were detected for age, BMI, LV-EDV, LV-mass, LVEDD, GWMS and LGE extent (Table 3). Right ventricular systolic function tended to be also reduced in those patients with depressed LV function, however, this difference did not reach statistical significance. In order to find out determinants of reduced LVEF (as measured by CMR) we performed simple linear regression analysis (Table 4 and Fig. 3): There was a significant decline of LVEF with increasing myocardial damage extent (as measured by LGE imaging). Decreased LVEF was also associated with increasing LV-EDV, LV mass and LVEDD. As expected, the highest coefficient of determination of LVEF was calculated for the parameter GWMS confirming that reduction in LVEF is due to the extent and severity of rWMA. Furthermore, higher age was associated with a decrease in systolic LV function. A meaningful multivariate regression analysis could not be performed due to the limited size of this cohort.

**Table 3:** Comparison of patients with normal and reduced LVEF as measured by CMR

	**All patients** **n = 15**	**Patients with LVEF ≥ 60%** **n = 5**	**Patients with LVEF <60%** **n = 10**	**p value**
**LVEF [%]**	52 (27 to 70)	65 (61 to 70)	42 (27 to 54)	**<0.001**
**Age [years]**	37 (11 to 56)	14 (11 to 26)	42 (23 to 56)	**0.004**
**BMI [kg/m^2^]**	23 (14 to 31)	19 (14 to 23)	23 (22 to 31)	**0.048**
**LV-EDV [ml]**	182 (94 to 230)	114 (94 to 212)	192 (129 to 230)	**0.047**
**LV-mass [g]**	117 (48 to 163)	70 (48 to 155)	119 (98 to 163)	**0.047**
**LVEDD [mm]**	52 (33 to 65)	46 (33 to 57)	55 (47 to 65)	0.09
**RVEF [%]**	56 (42 to 66)	65 (48 to 66)	54 (42 to 59)	0.10
**RV-EDV [ml]**	125 (104 to 213)	116 (104 to 213)	134 (104 to 180)	0.79
**GWMS**	0.19 (0 to 1.06)	0	0.34 (0 to 1.06)	**<0.001**
**LGE [%]**	13.0 (0 to 38.0)	0 (0 to 13.0)	17.4 (0 to 38.0)	**0.010**
**Total CK [U/L]**	1532 (310 to 28194)	3668 (2570 to 28194)	602 (310 to 3609)	**0.006**

Results are presented as median in addition to minimal and maximal values; LVEF = left ventricular ejection fraction; BMI = body mass index; LV-EDV = left ventricular end-diastolic volume; LVEDD = left ventricular end-diastolic diameter; RVEF = right ventricular ejection fraction; RV-EDV = right ventricular end-diastolic volume; GMWS = global wall motion score; LGE = late gadolinium enhancement; CK = creatinine kinase.

**Table 4:** Determinants of LVEF: simple linear regression analysis using log [LVEF] (intercept not shown)

**Variable**	**Coefficient ± SE**	**p value**
**LGE**	-4.67 ± 0.88	0.035
**LV-EDV**	-0.76 ± 0.10	0.003
**LV mass**	-0.87 ± 0.13	0.013
**Age**	-1.43 ± 0.17	0.003
**GWMS**	-9.44 ± 0.73	<0.001
**LVEDD**	-0.34 ± 0.06	0.028

LVEF = left ventricular ejection fraction; LGE = late gadolinium enhancement; LV-EDV = left ventricular end-diastolic volume; GMWS = global wall motion score; LVEDD = left ventricular end-diastolic diameter.

### Necessity of medical heart failure therapy

Clinical examination revealed skeletal muscle involvement in 12 patients (80.0%), however, at least minor degrees of skeletal muscle weakness and pathology were detected by EMG in all patients limiting their effort and exercise capacity, thereby potentially masking cardiac exercise-dependent symptoms. Only four patients (26.7%) were already taking heart failure medication (Table 1) although cardiac involvement including early or advanced DCM had been previously diagnosed in seven patients (46.7%). CMR examination revealed cardiac involvement in five more patients increasing the number of patients with abnormal left ventricles to 12 (80.0%; p = 0.019) with 10 of them (66.7%) having reduced LV systolic function. Therefore, these patients were regarded as in need for medical therapy (p = 0.009 compared to those patients already on therapy) considering the recommendations of the guidelines on heart failure therapy [9].

## Discussion

To the best of our knowledge, this is the first study subjecting a group of 15 patients with BMD (aged 11 years to 56 years) to comprehensive cardiological work-up including LGE imaging with CMR. Our results indicate that cardiac evaluation with CMR is more sensitive in detecting abnormal findings compared to ECG and conventional echocardiogpraphy, respectively. The findings of this study suggest that progressive myocardial damage is causing regionally accentuated loss in contractility, thereby resulting in progressive decrease in global LV systolic function. Furthermore, cardiac involvement in terms of myocardial damage in patients with BMD is predominantly beginning at the subepicardium of the inferolateral wall in the 3^rd ^decade of life with age-dependent increase in its extent. In addition, for the first time a substantial agreement between the presence of myocardial damage (as demonstrated by LGE imaging) and rWMA was found suggesting that a progressive reduction in LVEF is primarily due to the extent of myocardial damage.

### Incidence of cardiac involvement in BMD

Cardiac involvement has been described in patients with MD for decades by post-mortem analyses [15,16]. In previous studies, mainly echocardiographic and scintigraphic methods [4,15,17] were applied for cardiac evaluation of patients with MD: In BMD patients, cardiac involvement was found to be uncommon in patients aged <16 years, while more than 70% did demonstrate pathologic cardiac findings by the age of 40 years [4,17]. Our results are in accordance with these previous studies, since the only three patients without any pathological finding were aged 11 years to 14 years. However, this study extends previous knowledge, since we could demonstrate pathological findings (including reduced LVEF and/or presence of LGE) by CMR in all patients aged ≥23 years (n = 12; 80.0%) while conventional echocardiography revealed pathological results only in eight patients aged ≥23 years (53.3%; p = 0.040). The main advantage of CMR as compared to conventional echocardiography is the possibility to detect myocardial damage resulting in abnormal examinations in 12 patients.

### Method-based findings in BMD patients

Blood sample analysis for total CK revealed increased CK levels in all patients included to this study. Interestingly, higher CK-values were found in those with still preserved LV function. This finding is in accordance with previous studies suggesting that there is no correlation between cardiac disease severity and the degree of skeletal myopathy [4,17]. Surprisingly, elevated BNP levels were observed only in two of the ten patients with depressed LV function. One explanation for this observation may be that the degree of LV dysfunction is not severe enough to cause a rise in BNP [18]. Since BNP levels are supposed to also reflect rapid changes of pressure gradients in the ventricles [19,20], one may argue that the underlying pathophysiology in muscular dystrophy is mainly driven by continuous myocardial cell death (due to dystrophin absence or fragility) resulting secondarily in a continuous and adapted ventricular enlargement with progressive decrease of systolic function without quick changes in ventricular filling pressures.

In the past, ECG changes attracted great interest as sensitive signs of cardiac involvement in BMD patients. The typical ECG changes described in the literature were felt to reflect a reduction in electromotive forces in the posterobasal and contiguous lateral wall [15,21]. However, the shortcomings of a cardiac examination relying mainly on ECG findings are illustrated in this study: Only seven patients (46.7%) did demonstrate some ECG abnormalities with only four patients (26.7%) having those ECG changes that were described previously to be typical in muscular dystrophy. In comparison to ECG, 12 patients (80.0%) had evidence of cardiac involvement by CMR analysis.

The comparison of patients with normal and reduced LVEF confirmed previous results: patients with reduced LVEF were older, had larger and heavier hearts and demonstrated more rWMA. Left ventricular characteristics such as LV-EDV, LV mass and LVEDD were significantly associated with LVEF as shown by simple linear regression analysis. Interestingly, we found a substantial agreement between the presence of myocardial damage as demonstrated by LGE imaging and rWMA (kappa = 0.69). Since the highest coefficient of determination of LVEF was calculated for the parameter GWMS which depends on the extent and severity of rWMA which in turn is related to regionally pronounced myocardial damage, we conclude that the progressive reduction in LVEF is primarily due to the extent of myocardial damage.

### Pattern and pathophysiology of myocardial damage in BMD patients

In post mortem hearts, it has already been demonstrated that myocardial damage is starting from the epicardial third of the inferolateral wall with possible transmural extension in contiguous segments [16,22] and a genetical predilection of the posterobasal and lateral LV walls in patients with MD was suggested [21]. Our results obtained in the present study by state-of-the-art non-invasive CMR imaging confirm those previous results and impressively illustrate the tremendous progress in medicine: assessment of myocardial damage which could only be done by invasive ex vivo histopathological work-up at necropsy in the 1960s and 1970s, is today achieved by non-invasive in vivo CMR. Recently, Silva et al. reported their CMR findings in a small and considerably different collective (compared to ours) of only 10 young patients primarily suffering from DMD. They observed myocardial fibrosis in seven out of their 10 patients with the lateral wall being most commonly involved [23].

The detailed cellular and molecular mechanisms leading to myocardial contractile dysfunction in patients with MD are still to be elucidated. Preliminary animal studies suggest early alterations in cell metabolism and signal transduction in dystrophin-deficient hearts with a possible defect in the NO/cGMP pathway [24] and excessive intracellular Ca-signaling and ROS generation with breakdown of the mitochondrial membrane potential [25]. Interestingly, changes in regional myocardial metabolism have been documented at early disease stages, when rWMA or fibrosis were still absent [21]. Therefore, morphological changes in the myocardium of patients with MD are believed to be a consequence of the underlying genetically-determined metabolic and structural abnormalities which can be aggravated by additional mechanical stress.

### Relevance of experiences in myocarditis for cardiac involvement in muscular dystrophy?

**Figure 4:**
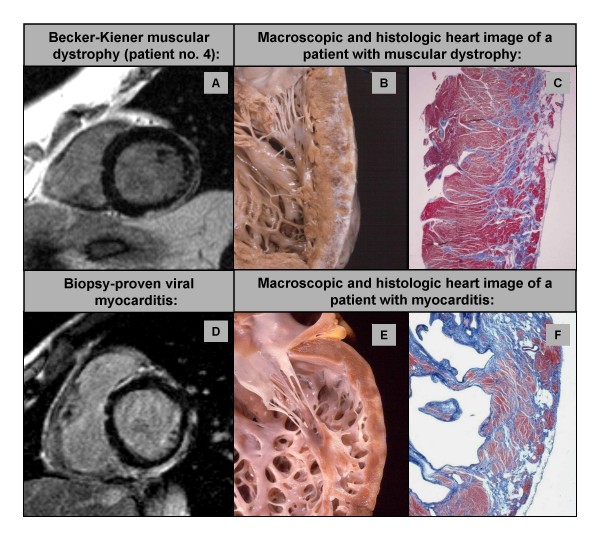
LGE short-axis image of patient no. 4 (A) with a subepicardial pattern of myocardial damage in the inferolateral wall highly resembling the myocardial damage pattern observed in patients with viral myocarditis (D). Exemplary macroscopic image of a different patient with muscular dystrophy (type Duchenne; B) and another different patient with chronic myocarditis (E) demonstrating the similarities in the pattern of myocardial damage: note the diffuse subepicardial fibrosis along the posterolateral LV free walls, with almost preserved wall thickness. Corresponding full thickness histologic slides (C and F): the extensive fibrous tissue replacement is typically confined to the outer-mid subepicardial layer of the LV free wall.

Interestingly, our group as well as other research groups have shown previously that this subepicardial pattern of myocardial damage which was detected predominantly in the posterolateral myocardial wall of patients with BMD is also a characteristic finding in biopsy-proven myocarditis [14,26,27]. This typical feature has become useful for non-invasive diagnosis of myocarditis by CMR (Fig. 4). Indeed, a final common pathway leading to myocardial damage in patients with MD and (entero-) viral myocarditis has been suggested previously [28,29]. In both diseases, progressive LV dysfunction is thought to be the result of dystrophin protein disruption [30-32]. Although cleavage of dystrophin has only been documented for coxsackievirus B3 [30,33], similar mechanisms for induction of myocardial damage can be envisaged also for other cardiotropic viruses. However, this needs further studies.

As we did not perform endomyocardial biopsies in our patients with BMD, we could not rule out that myocarditis was the ultimate cause of the observed posterolateral pattern of myocardial damage. However, due to the fact that almost all patients aged ≥23 years showed this pattern, and all patients had proof of genetically determined abnormalities of dystrophin protein, an additional infectious cause seems highly unlikely.

How can one explain that a presumably diffusely distributed dystrophin abnormality in the human heart results in a pattern of myocardial damage which is preferentially located in the subepicardial portions of the posterolateral wall? One possibility is that the genetically determined dystrophin damage is not diffusely distributed but accentuated in the posterolateral wall [16]. The other possibility is that a diffuse (genetically determined) disease process with ubiquitous alterations in cell metabolism and signal transduction precedes functional impairment [24,25] leading to myocardial damage preferentially in the posterolateral wall. Based on this hypothesis, the myocardial damage in the posterolateral wall could represent a non-specific cardiac phenotype in response to exaggerated mechanical stress in this region. The fact that a diversity of presumably diffuse diseases such as sarcoidosis, Chagas disease, Anderson-Fabry disease [34] or arrhythmogenic right ventricular cardiomyopathy [35] demonstrate the main damage in the posterolateral wall makes this yet unproven hypothesis attractive. Different pathophysiologies (such as myocardial inflammation or intracellular accumulation of storage products) may predispose the human heart to myocardial damage, which is amplified by increased mechanical stress and characterised by necrosis and/or fibrosis beginning in the inferolateral wall. Whether the inferolateral wall constitutes the "weakest cadet" of LV segments due to metabolic, structural or functional characteristics or otherwise has to withstand the highest mechanical stress during LV contraction and relaxation still has to be explored.

### Therapeutic and prognostic consequences of CMR results

Early cardiac evaluation including state-of-the-art non-invasive imaging of young patients with MD is required in order to identify those who will most likely benefit from early heart failure therapy. Timely onset of heart failure medication has been shown to result in beneficial ventricular remodelling with improvement in LV systolic function or at least in retardation of progressive cardiac dysfunction [6,7,36]. In our study group, heart disease had already been established by echocardiographic studies in seven patients, however, only four (26.7%) were taking heart failure medication. Obviously, the decision about putting a patient with MD on heart failure medication cannot only be based on clinical symptoms, since most patients suffer from skeletal myopathy and therefore avoid exaggerated physical activity, thereby masking potential symptoms. When considering the potential rapid progression of cardiac involvement in these patients and the proven beneficial effects of medical therapy [6,7,36], extensive and regular cardiac evaluations using CMR should become the standard of cardiac care for these patients. Recently published ACC/AHA guidelines on the diagnosis and treatment of heart failure suggest that heart failure therapy should be begun even in the absence of clinical symptoms if there is evidence of structural abnormalities and reduced LV function [8,9]. Compared to this standard, the majority of our patients were hitherto undertreated. We started medical heart failure therapy in those patients in this study who had a decreased LVEF (n = 10; 66.7%) independent of their symptoms. Whether patients who only show some amounts of LGE in spite of normal LVEF – as was the case in two patients – will also benefit from early medical therapy, has to be explored in future studies.

### Study limitations

The study size was small (with only 15 BMD patients) preventing further statistical analyses. However, considering the prevalence rate of this rare disease, it still constitutes a substantial number and no comparable CMR-based comprehensive studies focusing on LGE-imaging have been performed on a cohort of BMD patients previously, although there is a prior case report [37]. We acknowledge that new techniques such as CMR-based strain imaging [38] or echocardiography-based tissue-doppler and strain imaging [39] represent promising tools for early detection of subclinical LV dysfunction in MD patients. However, these techniques are still under investigation and do not belong to the established and clinically used armentarium of most centers. Although the value of these methods in comparison to CMR-based LGE imaging will be of great interest, for the purpose of this study only well accepted and widely established echocardiographc and CMR-based methods were applied.

## Conclusion

Cardiac evaluation with CMR is more sensitive in detecting abnormal findings in BMD patients compared to conventional echocardiogpraphy. Myocardial damage is predominantly beginning at the subepicardium of the inferolateral wall – similar to cardiomyopathy in viral myocarditis – with an age-dependent increase in its extent and a progressive reduction of LVEF. Based on the results of this study, regular CMR examinations in BMD patients older than 20 years may be useful in order to prevent the underdiagnosis of cardiac involvement and the delay in onset of beneficial heart failure medication.

## Abbreviations

BMD: Becker-Kiener muscular dystrophy; BNP: brain-natriuretic-peptide; CMR: cardiovascular magnetic resonance; CK: creatine kinase; CRP: C-reactive-protein; DCM: dilated cardiomyopathy; DMD: Duchenne muscular dystrophy; GWMS: global wall motion score index; LGE: late gadolinium enhancement; LV: left ventricular; LVEF: left ventricular ejection fraction; MD: muscular dystrophy; rWMA: regional wall motion abnormalities; TnI: troponin-I.

## Competing interests

The authors declare that they have no competing interests.

## Authors' contributions

AY designed the study, participated in the CMR exams, carried out the data and statistical analysis, and wrote the manuscript. HJG participated in the study design and carried out the neurological examinations. HB participated in the study design and the CMR exams and carried out the data analysis. HM participated in the analysis of the CMR data and reviewed the manuscript. GM participated in the analysis of the CMR data and reviewed the manuscript. CB participated in the evaluation of the study results and reviewed the manuscript. GT participated in the evaluation of the study results and reviewed the manuscript. ADS carried out the neurological examinations and reviewed the manuscript. ACL supervised the neurological examinations and reviewed the manuscript. US supervised the study and critically reviewed the manuscript.
